# A Maximum Likelihood Based Nonparametric Iterative Adaptive Method of Synthetic Aperture Radar Tomography and Its Application for Estimating Underlying Topography and Forest Height

**DOI:** 10.3390/s18082459

**Published:** 2018-07-30

**Authors:** Xing Peng, Xinwu Li, Changcheng Wang, Haiqiang Fu, Yanan Du

**Affiliations:** 1 School of Geosciences and Info-Physics, Central South University, Changsha 410083, China; hubeipx@csu.edu.cn (X.P.); haiqiangfu@csu.edu.cn (H.F.); 2 Key Lab of Digital Earth Sciences, Institute of Remote Sensing & Digital Earth, Chinese Academy of Sciences, Beijing 100094, China; lixw@radi.ac.cn; 3 Key Laboratory of Metallogenic Prediction of Nonferrous Metals and Geological Environment Monitoring, Ministry of Education, Central South University, Changsha 410083, China; 4 School of Geographical Sciences, Guangzhou University, Guangzhou 510006, China; yndu@gzhu.edu.cn

**Keywords:** underlying topography, forest height, IAA-ML, SAR tomography, P-band

## Abstract

Synthetic aperture radar tomography (TomoSAR) is an important way of obtaining underlying topography and forest height for long-wavelength datasets such as L-band and P-band radar. It is usual to apply nonparametric spectral estimation methods with a large number of snapshots over forest areas. The nonparametric iterative adaptive approach for amplitude and phase estimation (IAA-APES) can obtain a high resolution; however, it only tends to work well with a small number of snapshots. To overcome this problem, this paper proposes the nonparametric iterative adaptive approach based on maximum likelihood estimation (IAA-ML) for the application over forest areas. IAA-ML can be directly used in forest areas, without any prior information or preprocessing. Moreover, it can work well in the case of a large number of snapshots. In addition, it mainly focuses on the backscattered power around the phase centers, helping to detect their locations. The proposed IAA-ML estimator was tested in simulated experiments and the results confirmed that IAA-ML obtains a higher resolution than IAA-APES. Moreover, six P-band fully polarimetric airborne SAR images were applied to acquire the structural parameters of a forest area. It was found that the results of the HH polarization are suitable for analyzing the ground contribution and the results of the HV polarization are beneficial when studying the canopy contribution. Based on this, the underlying topography and forest height of a test site in Paracou, French Guiana, were estimated. With respect to the Light Detection and Ranging (LiDAR) measurements, the standard deviation of the estimations of the IAA-ML TomoSAR method was 2.11 m for the underlying topography and 2.80 m for the forest height. Furthermore, compared to IAA-APES, IAA-ML obtained a higher resolution and a higher estimation accuracy. In addition, the estimation accuracy of IAA-ML was also slightly higher than that of the SKP-beamforming technique in this case study.

## 1. Introduction

The biophysical parameters of forest, such as the underlying topography and forest height, are important when quantifying the above-ground biomass (AGB) [1,2,3,4]. When used with long-wavelength data such as L-band and P-band radar, the synthetic aperture radar (SAR) technique has been proven to be an invaluable tool for estimating biophysical parameters, owing to its capability of penetrating deep into forests.

The retrieval of the underlying topography and forest height with the SAR technique involves the vertical location detection of the ground and canopy scattering phase centers. Since the SAR tomography (TomoSAR) technique is able to achieve three-dimensional imaging, it can provide the vertical structure of the observed scene, allowing us to recognize these scattering phase centers. The idea behind the concept of TomoSAR is that it forms an additional aperture along the vertical direction by combining multi-baseline SAR acquisitions [5,6]. This makes it possible for TomoSAR to achieve the vertical resolution and discriminate the different scatterers within one resolution cell. As a result, TomoSAR is now regarded as a viable tool for underlying topography and forest height estimation.

The key to estimating the underlying topography and forest height by TomoSAR is the tomographic focusing. In fact, the tomographic focusing can be considered as a spectral estimation problem and several spectral estimation approaches have been developed to solve the problem. These estimators can be divided into three groups: compressed sensing (CS) based methods [7,8,9,10,11,12,13,14], parametric spectral estimation methods [15,16,17,18,19,20] and nonparametric spectral estimation methods [21,22,23,24,25,26,27,28]. The CS-based methods, such as wavelet-based CS [9,10,11,12] and sum of Kronecker product decomposition (SKP [13])-based CS (SKP-CS [14]), can obtain a high resolution along the vertical direction, without any prior information. However, the computational consumption is their main drawback and a preprocessing step is required to search for a sparse expression for forested areas. The parametric spectral estimation methods, such as multiple-signal classification [15,16] and weighted subspace fitting [17], can attain a high vertical resolution. However, these methods require us to know the number of scatterers in each resolution cell, which is difficult over forest scenes as the forest canopy response consists of numerous random scattering contributions. The nonparametric spectral estimation methods, such as beamforming, Capon and IAA-APES [28], are robust to focusing artifacts and do not require any prior information or any preprocessing, avoiding the disadvantages of the first two groups of methods. However, some of the nonparametric spectral estimation methods, such as beamforming and Capon, obtain a low resolution. Although IAA-APES can obtain the highest resolution among all the nonparametric group of algorithms, it only tends to work well with a small number of snapshots. In practice, over forest areas, it is general to exploit the phase and amplitude information contained in the covariance matrix to perform tomographic focusing. In addition, we commonly take a large number of snapshots (much greater than the number of images) to estimate the covariance matrix, suppressing the strong coherent noise. Thus, it is necessary to develop a high-resolution nonparametric method suitable for use with a large number of snapshots.

To solve this problem, the nonparametric iterative adaptive approach based on maximum likelihood estimation (IAA-ML) is proposed in this paper. IAA-ML employs the negative log-likelihood function of multiple snapshot observations as the cost function. It can be directly used in forest areas, without any prior information or preprocessing. Compared to IAA-APES, it can work well in the case of a large number of snapshots. Moreover, it mainly focuses on the backscattered power around the ground and canopy scattering phase centers, helping to detect their location. In particular, for the application of array processing, Yardibi et al. [29,30] demonstrated that IAA-ML can achieve a higher resolution than IAA-APES when the number of snapshots is larger than the number of acquisitions. Thus, IAA-ML is a better alternative for the forest application of TomoSAR.

The rest of this paper is organized as follows. Section 2 gives a brief introduction to the TomoSAR imaging model and explains the IAA-ML methodology in SAR tomography. Section 3 then describes and analyzes the results of the simulated experiments. Section 4 describes the study area and datasets. The results of the underlying topography and forest height estimation by the IAA-ML approach with real data are also presented and the results are evaluated with LiDAR measurements. A further discussion about the difference between IAA-ML and the other methods (IAA-APES and SKP-beamforming) is given in Section 5. Finally, our conclusions are drawn in Section 6.

## 2. Methodology

### 2.1. Overview of the TomoSAR Imaging Model

We assume that *N* SAR images are available over the area of interest at slightly different viewing angles *θ*. For the *n*th image after coregistration, deramping and phase calibration with respect to a common master image, the focused complex value *y_n_*(*l*) at an arbitrary pixel(*x*_0_*,y*_0_) can be expressed as [6]:

(1) $$y_{n}\left(l\right)=\int \gamma_{s}\left(l\right)\exp\left(-j2\pi \xi_{n}s\right)ds$$

where l=1,⋯,L indicates one of the *L* independent realizations of the signal acquisition. In other words, *L* means the number of snapshots or looks. $\gamma_{s}\left(l\right)$ represents the complex scattering coefficients along the elevation direction and *j* is the complex number unit. $\xi_{n}$ is the spatial frequency related to the perpendicular baseline $b_{n}$, the slant range between the master track and the scatterer *r* and wavelength λ. It is written as [6]:

(2) $$\xi_{n}=\frac{-2b_{n}}{\lambda r}$$

In fact, the continuous-space system model of Equation (1) can be regarded as a randomly sampled Fourier transform of $\gamma_{s}\left(l\right)$. Thus, we can define $\rho_{s}=\frac{\lambda r}{2\Delta b}$ as an inherent Rayleigh resolution for the elevation, where Δb is the total baseline span. The transformation factor between the elevation *s* and the height *z* perpendicular to the horizontal plane can be expressed as:

(3) z=s×sinθ

The imaging model (Equation (1)) can thus be expressed as [6,17]:

(4) $$y_{n}\left(l\right)=\int \gamma_{z}\left(l\right)\exp\left(jk_{z}\left(n\right)z\right)dz$$

where $k_{z}\left(n\right)=\frac{4\pi b_{n}}{\lambda rsin\theta }$ is the vertical wavenumber of the *n*th image with respect to the master track. $\gamma_{z}\left(l\right)$ denotes the complex scattering coefficients along the vertical direction.

Through discretizing the continuous reflectivity function $\gamma_{z}\left(l\right)$ with D intervals, the system model (Equation (4)) can be approximately written as [6,17]:

(5) y(l)=Ax(l)+e(l)

where *y*(*l*) is the measurement vector of *N* elements with $y\left(l\right)=\left[y_{1}\left(l\right),y_{2}\left(l\right),\cdots ,y_{N}\left(l\right)\right]$; x(l) is the unknown discrete reflectivity vector with *D* elements with $x_{d}\left(l\right)=\gamma_{z_{d}}\left(l\right)$; $z_{d}\left(d=1,\cdots ,D\right)$ represents the discrete height position; *e*(*l*) is the noise vector that contains *N* elements; and *A* is an N×D mapping matrix with $A=\left[a_{1},\cdots ,a_{D}\right]$. The steering vector $a_{d}$ is given by:

(6) $$a_{d}={\left[\exp\left(jk_{z}\left(1\right)z_{d}\right),\cdots ,exp\left(jk_{z}\left(N\right)z_{d}\right)\right]}^{T}$$

where ${\left(\cdot \right)}^{T}$ represents the transpose operator of a vector or a matrix.

### 2.2. IAA-ML TomoSAR Method

In this section, we propose the IAA-ML TomoSAR method for application in forested areas. IAA-ML is a kind of nonparametric iterative adaptive approach based on maximum likelihood estimation and is aimed at estimating the backscattered power around the scattering phase centers. 

We let P be a D×D diagonal matrix, whose diagonal contains the power of reflectivity at each vertical location. These diagonal elements are the parameters of interest. 

For the TomoSAR imaging model (Equation (5)), the covariance matrix of *y*(*l*) can be given by [29,30,31]:

(7) $$R=E\left(y\left(l\right)y^{*}\left(l\right)\right)≜APA^{*}$$

where E(·) is the expectation operator and ${\left(\cdot \right)}^{*}$ represents the conjugate transpose operator of a vector or a matrix.

The IAA-ML algorithm minimizes the negative log-likelihood function of the observations. The cost function of Equation (5) is given by [32,33]:

(8) $$f=\ln\left|R\right|+\frac{1}{L}\overset{L}{\underset{l=1}{\sum }}y^{*}\left(l\right)R^{-1}y\left(l\right)$$

Setting the first derivative of Equation (8) with respect to $p_{d}$ to zero, gives [29,30,31]:

(9) $$p_{d}=\frac{a_{d}^{*}Q_{d}{}^{-1}\left(\Gamma -Q_{d}\right)Q_{d}^{-1}a_{d}}{{\left(a_{d}^{*}Q_{d}^{-1}a_{d}\right)}^{2}}$$

where $\Gamma =\frac{1}{L}\sum_{l=1}^{L}y\left(l\right)y{\left(l\right)}^{*}$ is the sample covariance matrix. $Q_{d}$ is the covariance matrix of the interference and noise, with $Q_{d}=R-p_{d}a_{d}a_{d}^{*}$.

In practice, $p_{d}$ may be negative. Therefore, the non-negativity of the power estimates is enforced by setting the negative estimates to zero at each iteration. Moreover, we use the matrix inversion lemma to replace $Q_{d}$ with *R*. Accordingly, the IAA-ML power estimate is obtained as [29,30,31]:

(10) $$p_{d}=max\left(0,p_{d}+\frac{a_{d}^{*}R^{-1}\left(\Gamma -R\right)R^{-1}a_{d}}{{\left(a_{d}^{*}R^{-1}a_{d}\right)}^{2}}\right)$$

By inspecting Equations (7) and (10), parameter ${\hat{p}}_{d}$ needs *R* and *R* also requires ${\hat{p}}_{d}$. Accordingly, this approach must operate in an iterative way, as shown in Table 1. Note that the sorting procedure is used to drive the estimates for the potential source-free locations to zero. The iterative process is terminated when the current estimates of *P* for the last two iterations are almost constant. After obtaining the reflectivity power estimates ${\left\{p_{d}\right\}}_{d=1}^{D}$, the ground and canopy scattering phase centers can be determined by detecting the coordinates of the two largest local maxima of the reflectivity power.

## 3. Numerical Simulated Experiments

Based on the acquisition parameters described in Section 4 (see Table 2 and Table 3), simulated experiments were performed to investigate the advantages of the IAA-ML TomoSAR method.

For forested areas, there are a variety of scattering mechanisms, including surface scattering from the ground, double bounce scattering between the ground and tree trunks, double bounce scattering between the ground and the forest canopy and volume scattering from the forest canopy. The phase centers of the first two kinds of scattering mechanisms are fixed at the ground and the phase center of volume scattering is located in the middle of the canopy [8,11]. Thus, we usually regard the backscattered power as being contributed from both the ground and canopy. According to the aforementioned analysis, we assumed two sets of backscattered signals with different phase center location differences (30 m and 8 m, respectively). Both sets of backscattered signals had two components. One component represented the contribution from the canopy, with a high location and a wide angular spread, while the other component represented the ground contribution, with a low location and a narrow angular spread. Moreover, we considered two kinds of baseline distributions: uniform and non-uniform. The uniformly distributed baselines are shown in Table 3. As for the non-uniform distribution, the minimum and maximum baselines were both based on Table 3, ensuring the same Rayleigh resolution. In addition, the number of snapshots was 256 and the signal-to-noise ratio (SNR) was 20 dB. We let *t* be the power ratio of ground to canopy. When t>1, the ground contribution dominates; when t=1, the ground contribution is equal to the canopy contribution; and when t<1, the canopy contribution dominates. The tomographic inversion was carried out in the different simulated experiments on the extent of the elevation profile.

(1) We investigated the reconstruction performance of IAA-APES and IAA-ML for two sets of simulated signals with different baseline distributions.
(2) The reconstruction performance between IAA-APES and IAA-ML was then investigated for the two sets of simulated signals with different power ratios of ground to canopy.
(3) The resolution capability of IAA-ML was investigated in terms of detecting the two phase centers.

**Figure 1:**
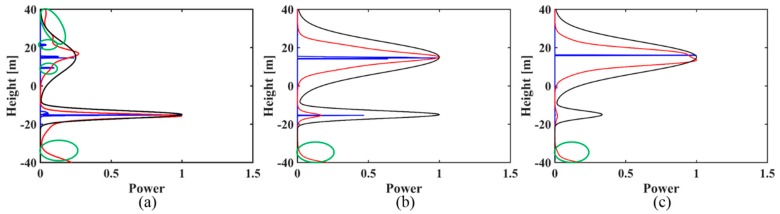
The reconstructed reflectivity profiles obtained by the IAA-APES and IAA-ML TomoSAR methods for the simulated signal (30 m phase center location difference) with different power ratios of ground to canopy in the case of uniformly distributed baselines: (**a**) *t* > 1; (**b**) *t* = 1; (**c**) *t* < 1. The black solid lines are the normalized simulated profiles. The red solid lines are the reconstructed reflectivity profiles of IAA-APES. The blue solid lines are the reconstructed reflectivity profiles of IAA-ML.

**Figure 2:**
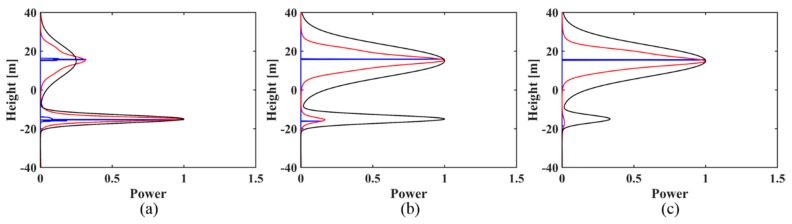
The reconstructed reflectivity profiles obtained by the IAA-APES and IAA-ML TomoSAR methods for the simulated signal (30 m phase center location difference) with different power ratios of ground to canopy in the case of non-uniformly distributed baselines: (**a**) *t* > 1; (**b**) *t* = 1; (**c**) *t* < 1. The black solid lines are the normalized simulated profiles. The red solid lines are the reconstructed reflectivity profiles of IAA-APES. The blue solid lines are the reconstructed reflectivity profiles of IAA-ML.

**Figure 3:**
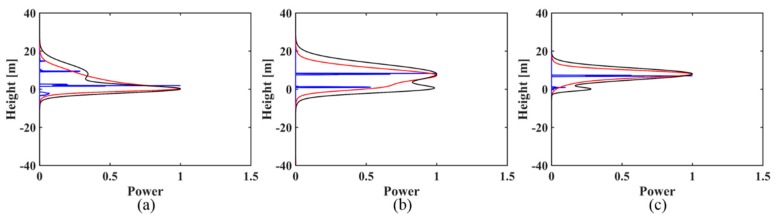
The reconstructed reflectivity profiles obtained by the IAA-APES and IAA-ML TomoSAR methods for the simulated signal (8 m phase center location difference) with different power ratios of ground to canopy in the case of uniformly distributed baselines: (**a**) *t* > 1; (**b**) *t* = 1; (**c**) *t* < 1. The black solid lines are the normalized simulated profiles. The red solid lines are the reconstructed reflectivity profiles of IAA-APES. The blue solid lines are the reconstructed reflectivity profiles of IAA-ML.

**Figure 4:**
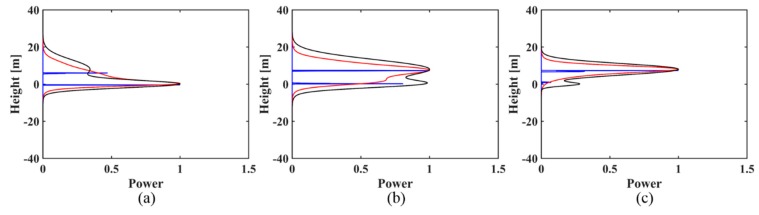
The reconstructed reflectivity profiles obtained by the IAA-APES and IAA-ML TomoSAR methods. For the simulated signal (8 m phase center location difference) with different power ratios of ground to canopy in the case of non-uniformly distributed baselines: (**a**) *t* > 1; (**b**) *t* = 1; (**c**) *t* < 1. The black solid lines are the normalized simulated profiles. The red solid lines are the reconstructed reflectivity profiles of IAA-APES. The blue solid lines are the reconstructed reflectivity profiles of IAA-ML.

**Figure 5:**
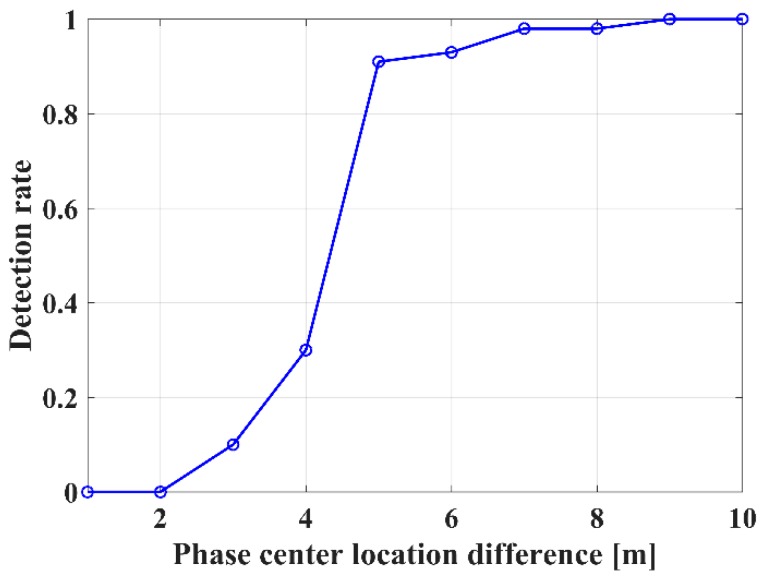
Detection rate of the ground and canopy phase centers with the IAA-ML method.

Based on the above simulations, some observations can be made:

(1) For the two sets of simulated signals, IAA-ML has much narrower main lobes than IAA-APES for all cases and it is aimed at estimating the backscattered power around the phase centers.
(2) For the simulated signal with two backscattering phase centers of 15 m and −15 m in the case of uniformly distributed baselines (as shown in Figure 1), when the ground contribution does dominate, that is, *t* > 1, then both the IAA-APES and IAA-ML estimators can successfully obtain the canopy and ground phase center information, including the location and power estimation, although some sidelobes exist (green circles in Figure 1). When the canopy power increases to the same level as the ground power (*t* = 1), the two methods can accurately reconstruct the canopy phase center information but show a degraded performance in detecting the ground contribution as the amplitude estimate deviates greatly from the true value, especially the result of IAA-APES (Figure 1b). When the canopy contribution dominates (*t* < 1), the two estimators can only retrieve the canopy phase center information and fail to recognize the ground scattering phase center (Figure 1c). As for the non-uniformly distributed baselines (see Figure 2), the two estimators show a similar reconstruction performance to the uniform case but with fewer sidelobes.

(3) For the simulated signal with two backscattering phase centers of 0 m and 8 m, in both the case of the uniformly distributed baselines and in the case of the non-uniformly distributed baselines, the IAA-ML estimator can successfully discriminate the canopy and ground phase centers under three kinds of ground to canopy power ratios (as shown in Figure 3 and Figure 4), although there is some bias for the height and amplitude estimation. However, IAA-APES can only detect the canopy scattering phase center and it fails to recognize the ground scattering phase center in these cases. When the canopy contribution dominates (*t* < 1), the IAA-ML method shows a decreased detection capability for the ground phase center (Figure 3c and Figure 4c).

(4) From Figure 5, IAA-ML can detect the two phase centers, even for a location difference of only 5 m (with a detection rate of over 90%).

Based on the above observations, it is found that both the IAA-ML and IAA-APES estimators can work with the uniformly and non-uniformly distributed baselines. Moreover, their reconstruction performance is closely related to the power ratio of ground to canopy. When the ground contribution does dominate, both estimators can accurately retrieve the ground scattering phase center. However, with the increase of the canopy contribution, both estimators show a degraded performance in detecting the ground phase center, especially when the canopy contribution dominates. In addition, IAA-ML obtains a higher elevation resolution and better focusing than IAA-APES, which is beneficial for the extraction of the underlying topography and forest height.

## 4. Real-Data Experiments and Results

A real airborne SAR dataset was applied in TomoSAR to obtain the underlying topography and forest height, investigating the feasibility and effectiveness of IAA-ML in comparison to LiDAR measurements.

### 4.1. Study Area and Dataset

**Figure 6:**
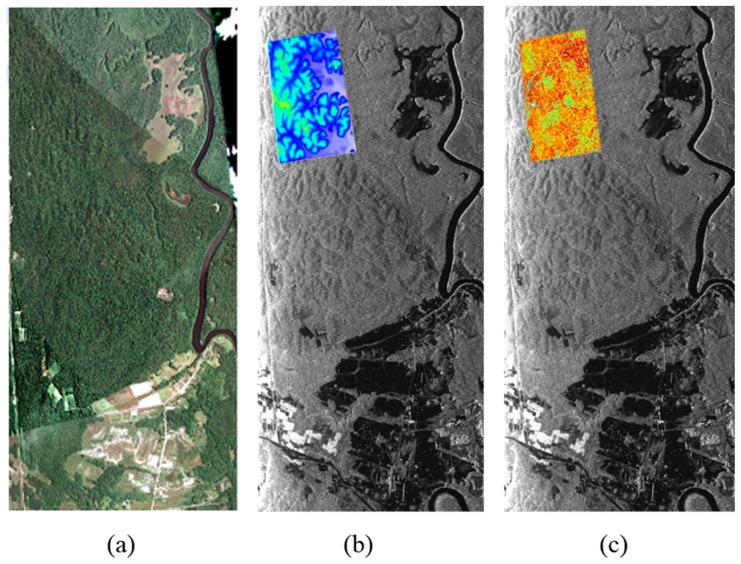
(**a**) Optical image of the study area. (**b**) SAR intensity map of the study area, with the LiDAR. Digital terrain model (DTM) overlaid on the SAR intensity map. (**c**) SAR intensity map of the study area, with the LiDAR canopy height model (CHM) overlaid on the SAR intensity map.

The study area is Paracou, French Guiana, which is an important experimental site for tropical rain forest (marked by the red rectangle in Figure 6a). The climate in this area is hot and rainy, with annual precipitation of about 2980 mm and an annual mean temperature of about 26 °C. The topography in the study site is fairly flat and the elevation ranges from 5 m to 50 m [34]. The forest types include both primitive forest and natural secondary forest restored after the destruction of logging, interference and other natural factors. There are various tree species, such as *Leguminosae*, *Chrysobalanaceae* and *Euphorbiaceae* [35]. The forest vertical structure is complex, with both primary forest and natural secondary forest and the tree height varies from 20–40 m [34].

A stack of six fully polarimetric focused P-band SAR images over the study area was used to demonstrate the potential of IAA-ML. This dataset was acquired by the ONERA SETHI airborne system (which was funded by the European Space Agency (ESA) TropiSAR 2009 campaign) on 24 August 2009. Some critical preprocessing steps have already been applied to this dataset, including coregistration and flat-earth phase removal. There are six repeat observation orbits and each one is parallel to the others. Moreover, the orbits are uniformly distributed along the vertical direction. The distance between two adjacent orbits is about 15.24 m. In addition, all the images were acquired within a timeframe of two hours. Thus, temporal decorrelation can be ignored.

The range resolution is 1.0 m and the azimuth resolution is 1.245 m. The incidence angle ranges from 25° to 60° from the near range to the far range [34]. Details of the parameters of the SETHI airborne system and the baseline information for the interferometric synthetic aperture radar (InSAR) pairs are listed in Table 2 and Table 3, respectively.

The LiDAR digital terrain model (DTM) and canopy height model (CHM) over Paracou were provided by the French Agricultural Research Center for International Development (CIRAD) and the Guyafor Project and were used to validate the performance of the tomographic estimators. These data were obtained by the ALTOA system in April 2009, with a flight height of between 120 m and 220 m, covering a small part of the SAR image, as shown in Figure 6b,c. The coverage is about 1.5 km along the range direction and 3.0 km in the azimuth direction. The LiDAR data were generated from point cloud data with a spatial resolution of 1 m [35].

### 4.2. Results and Analysis

#### 4.2.1. Tomograms of the Selected Azimuth Profiles

**Figure 7:**
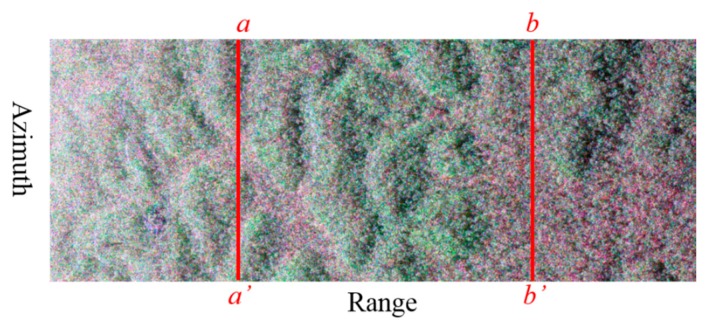
The two selected azimuth profiles (red solid lines *aa*’ and *bb*’) on the Pauli SAR image.

Because the vertical wavenumbers (*k_z_*(*n*)) are different in the near and far ranges for the airborne SAR data, two azimuth profiles (the red solid lines shown in Figure 7), located at the near range (line *aa*’) and the far range (line *bb*’), respectively, were selected as an example to undertake tomographic focusing, in order to further demonstrate the feasibility of IAA-ML. An estimation window of 39 × 39 pixels (slant range/azimuth) was used to estimate the covariance matrix. This indicates that the number of snapshots is much greater than the number of images (1521 vs. 6).

**Figure 8:**
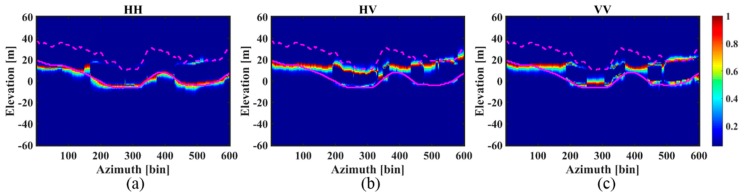
The tomograms of profile *aa*’ estimated by the IAA-ML method for the three different polarimetric channels: (**a**) HH; (**b**) HV; (**c**) VV. The pink dotted and solid lines represent DTM + CHM and DTM, respectively.

**Figure 9:**
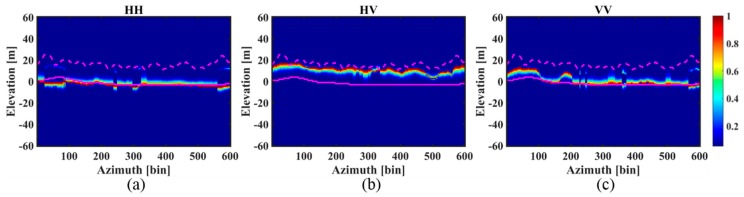
The tomograms of profile *bb*’ estimated by the IAA-ML method for the three different polarimetric channels: (**a**) HH; (**b**) HV; (**c**) VV. The pink dotted and solid lines represent DTM + CHM and DTM, respectively.

Figure 8 and Figure 9 respectively represent the tomograms of the three polarimetric channels for the *aa*’ and *bb*’ azimuth profiles. To allow a comparison, the corresponding LiDAR DTM and CHM based on DTM (DTM + CHM) are superimposed on the graphs. Similar observations can be made for the results of the two profiles: (1) For the HH polarization, IAA-ML successfully detects all the ground scattering phase centers, agreeing almost entirely with the LiDAR DTM but it only recognizes some of the canopy scattering phase centers. (2) For the HV polarization, IAA-ML detects all the canopy scattering phase centers, while it fails to recognize the ground scattering phase centers. Moreover, since the canopy scattering phase center location does not represent the height of the tree tops, the LiDAR CHM based on DTM is slightly higher than the canopy scattering phase center but they show a similar wave trend. (3) For the VV polarization, IAA-ML detects some of the ground scattering phase centers and some of the canopy scattering phase centers. 

The above observations suggest that the backscattered power of the HH polarimetric channel is mainly concentrated on the ground, the backscattered power of the HV polarimetric channel is mainly focused on the canopy and the backscattered power of the VV polarimetric channel centers on the ground in some parts and the canopy in other parts. This finding is in accordance with the result in [1] and it tells us that the result of the HH polarization is suitable for estimating the ground contribution and the result of the HV polarization is suitable for obtaining the canopy contribution. 

#### 4.2.2. Underlying Topography Estimation

**Figure 10:**
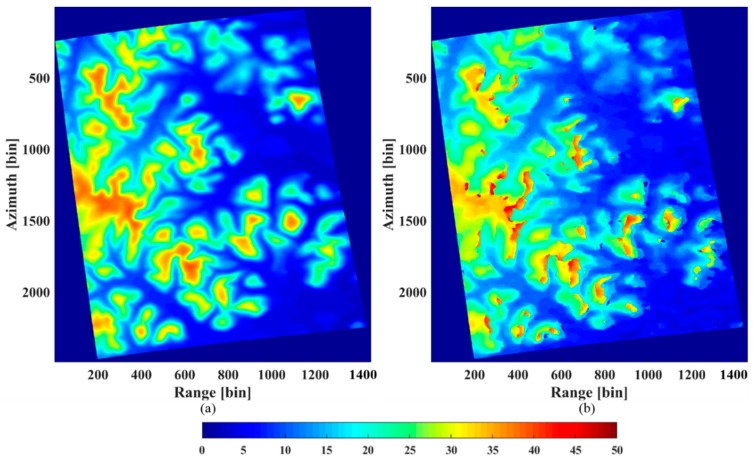
(**a**) The LiDAR DTM. (**b**) The underlying topography estimated by the IAA-ML TomoSAR method.

From the analysis in Section 4.2.1, we can estimate the underlying topography from the peaks of the tomograms in the HH polarization. In order to compare the estimation with the LiDAR DTM (Figure 10a), the underlying topography was inverted for the same coverage. The result is displayed in Figure 10b). Clearly, the estimated topography is consistent with the LiDAR DTM, although some bias does exist.

**Figure 11:**
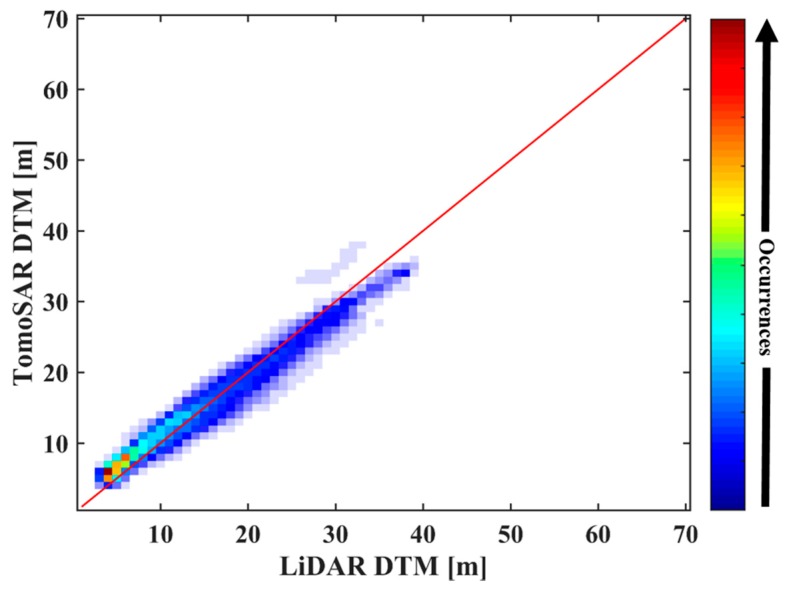
Joint distribution between the LiDAR DTM and the ground height estimated by the IAA-ML TomoSAR method.

A further comparison is given by the agreement analysis between the LiDAR DTM and the estimated result obtained by the IAA-ML TomoSAR method, as shown in Figure 11. The plot shows the joint distribution of the ground height, which is a normalized 2-D histogram counting the occurrence of the height combinations. It is evident that the consistency is high as the joint distribution of the ground heights locates at the diagonal (the red line in Figure 11) and its neighborhood.

Table 4 lists the mean (|TomoSAR−Liadar|) and the standard deviation (std.) of the underlying topography estimated by IAA-ML with respect to the LiDAR data, which are 1.76 m and 2.11 m, respectively. This suggests that the underlying topography estimated by the IAA-ML TomoSAR method is reliable.

#### 4.2.3. Forest Height Estimation

**Figure 12:**
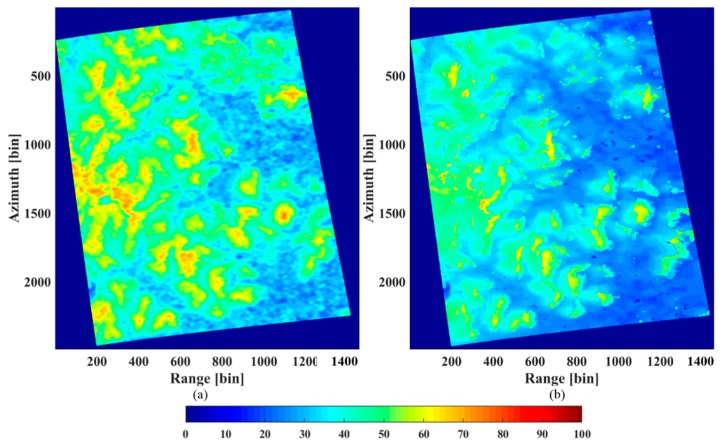
(**a**) The LiDAR CHM+DTM. (**b**) The canopy scattering phase center height estimated by the IAA-ML TomoSAR method.

The forest height can be obtained from the height difference between the canopy top height and the ground height. The ground height was estimated with the results of the HH polarization. From the analysis in Section 4.2.1, the height of the canopy scattering phase centers can be estimated from the tomograms in the HV polarization. Figure 12b shows the estimated canopy scattering phase center heights. Since the canopy scattering phase center location is not the tree top position, the estimated height is clearly lower than the LiDAR DTM based on CHM (Figure 12a).

**Figure 13:**
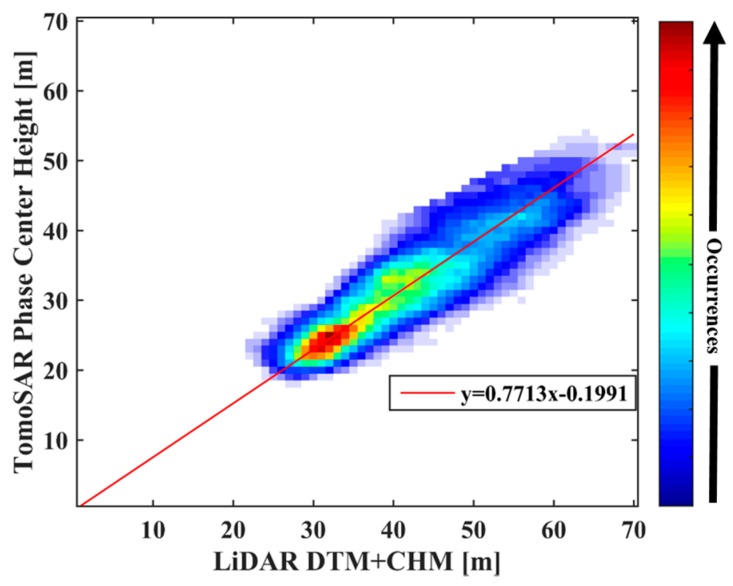
Correlation analysis between the LiDAR DTM + CHM and the phase center height estimated by the IAA-ML TomoSAR method.

Figure 13 shows the scatter plot between the canopy scattering phase center height from the IAA-ML estimator and the LiDAR CHM based on DTM, where it is found that they are positively correlated (0.7713). Note that the reference elevation is the sea level. According to this linear relationship, the tree top height can be obtained by calibrating the estimated phase center height.

**Figure 14:**
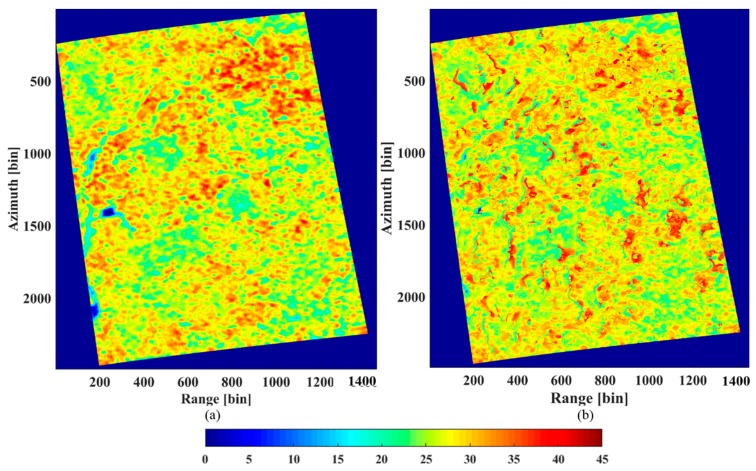
(**a**) The LiDAR CHM. (**b**) The TomoSAR CHM estimated by the IAA-ML TomoSAR method.

**Figure 15:**
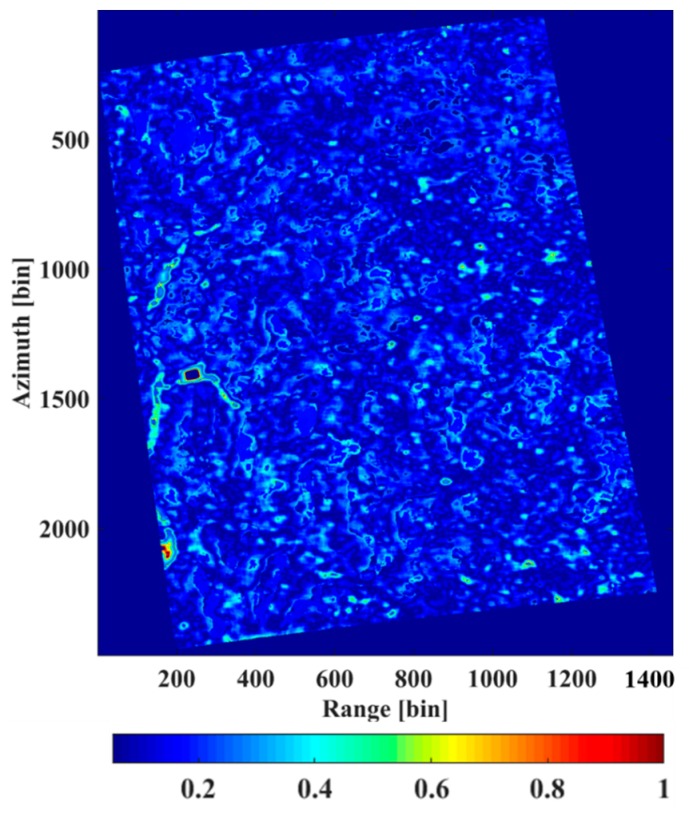
Relative error between the LiDAR CHM and the estimated forest height obtained by the IAA-ML method.

Figure 14 shows the LiDAR CHM and the forest height retrieved from the IAA-ML method with the HV polarimetric data. We can observe that the tree height varies from approximately 20 m to 40 m, which is consistent with the ground truth for this tropical forest area. For the vast majority of the region, the IAA-ML estimator shows a similar result to the LiDAR CHM. For the remaining part, some bias occurs due to the influence of several factors, such as noise and estimation error. Considering the reference elevation as the ground surface, the relative error was calculated as $|h_{tomo}-h_{lidar}|/h_{lidar}$, where $h_{tomo}$ is the estimated height obtained by the IAA-ML TomoSAR method and $h_{lidar}$ is the LiDAR measurement [24]. The relative error between the LiDAR CHM and the estimated forest height obtained by the IAA-ML method is shown in Figure 15 and the average relative error is 11.05%.

**Figure 16:**
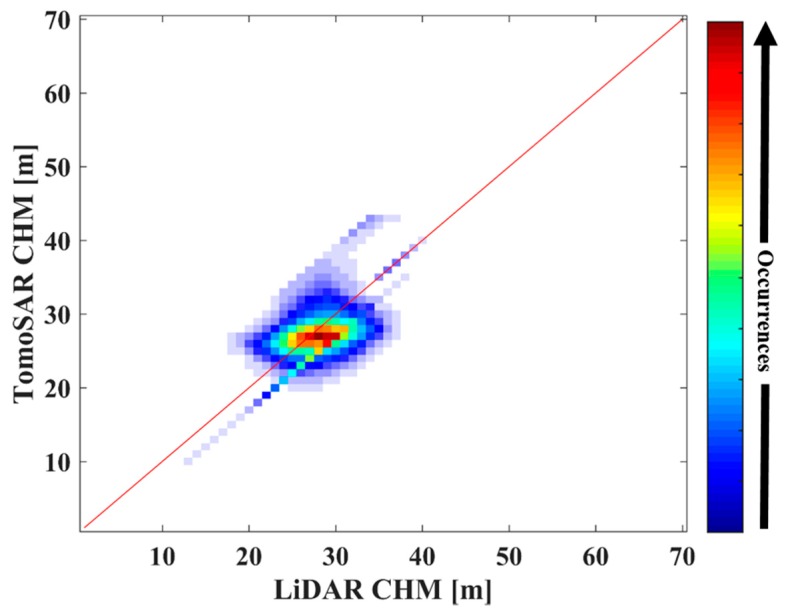
Joint distribution between the LiDAR CHM and the forest height estimated by the IAA-ML TomoSAR method.

Moreover, the corresponding consistency is analyzed in Figure 16, where it can be seen that the joint distribution of the forest height is centered around the diagonal.

In addition, a precise evaluation is given in Table 5. With regard to the LiDAR CHM, the standard deviation of the tree height estimated by the IAA-ML TomoSAR method is assessed as about 2.80 m. This also indicates that the IAA-ML TomoSAR method performs well in forest height estimation.

From the above analysis, it can be seen that the IAA-ML TomoSAR method can obtain reliable underlying topography and forest height estimations over forest areas with a P-band dual-polarimetric SAR dataset (HH and HV). However, the estimation error of the tree height is much larger than that of the underlying topography. This is because the tree height is retrieved from the difference between the tree top elevation and ground elevation. As there is some bias in both the tree top height and ground height, this results in error accumulation in the tree height estimation.

## 5. Discussion

**Figure 17:**
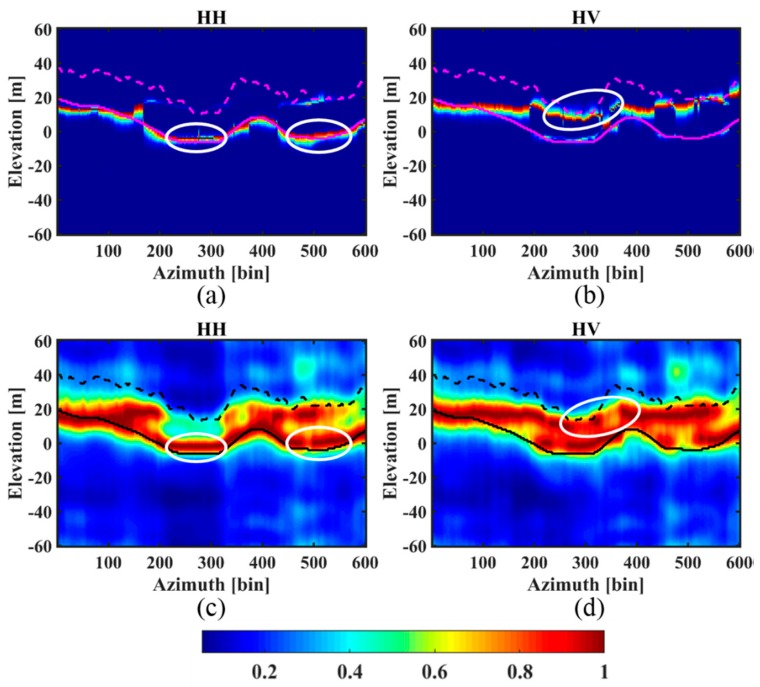
The estimated tomograms of the selected azimuth profile *aa*’ for the two TomoSAR estimators: (**a**) IAA-ML in HH polarization; (**b**) IAA-ML in HV polarization; (**c**) IAA-APES in HH polarization; (**d**) IAA-APES in HV polarization. The red and black solid lines represent the LiDAR DTM. The red and black dotted lines represent the LiDAR DTM + CHM.

**Figure 18:**
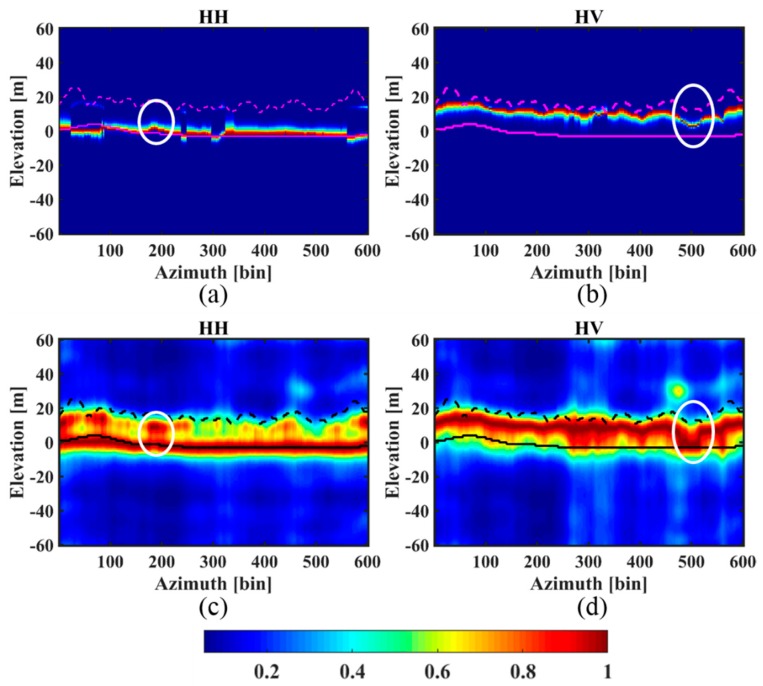
The estimated tomograms of the selected azimuth profile *bb*’ for the two TomoSAR estimators: (**a**) IAA-ML in HH polarization; (**b**) IAA-ML in HV polarization; (**c**) IAA-APES in HH polarization; (**d**) IAA-APES in HV polarization. The red and black solid lines represent the LiDAR DTM. The red and black dotted lines represent the LiDAR DTM + CHM.

In order to further demonstrate the performance of the IAA-ML TomoSAR method, IAA-APES was also used to undertake tomographic focusing at the same azimuth profiles (see the red solid lines *aa*’ and *bb*’ shown in Figure 7). The same parameters (estimation window, height range and height sample interval) were used in the IAA-APES method. Figure 17 and Figure 18 show the tomograms, that is, the backscattered power along the vertical direction, reconstructed by IAA-ML and IAA-APES for the *aa*’ and *bb*’ profiles, respectively. Meanwhile, we resampled the LiDAR measurements onto the SAR slant range/azimuth coordinates for the validation.

From Figure 17 and Figure 18, it can be seen that the backscattered power distribution estimated by IAA-APES is also related to the polarization mode. The power focuses on the ground in the HH polarization and the canopy in the HV polarization, respectively. However, the spectrum of the IAA-ML method is much narrower than that of the IAA-APES approach. This tells us that when the height of a scatterer changes slightly, IAA-ML is sensitive to this change but IAA-APES is not. In other words, IAA-ML obtains a higher resolution than IAA-APES in the TomoSAR forest application. In particular, for the segments marked by the white circles in Figure 17 and Figure 18, the IAA-ML estimates are much closer to the LiDAR data than the IAA-APES estimates. The above analysis confirms that the IAA-ML TomoSAR method can obtain a higher resolution than the IAA-APES approach over forest areas. 

Furthermore, the IAA-APES estimator was also applied to estimate the underlying topography with the results in HH polarization and forest height with the results in HV polarization over the study area. We calculated the standard deviation of the underlying topography and forest height obtained by the IAA-APES estimator with respect to the LiDAR data, as shown in Table 6, which are both larger than the values for the IAA-ML approach (2.57 m vs. 2.11 m and 3.29 m vs. 2.80 m). This indicates that the IAA-ML TomoSAR method obtains a higher estimation accuracy than the IAA-APES approach in underlying topography and forest height estimation over forest areas.

In addition, we also applied the SKP-beamforming technique to obtain the underlying topography and forest height with fully polarimetric data. The standard deviation of the SKP-beamforming technique is 2.41 m for the underlying topography and 3.00 m for the forest height, which are both slightly larger than the results of IAA-ML. 

## 6. Conclusions

In this paper, we have proposed the IAA-ML TomoSAR method, which is an iterative adaptive approach based on maximum likelihood estimation. IAA-ML allows reflectivity profile reconstruction in forested areas along the elevation direction and can discriminate different scatterers with a high accuracy.

By carrying out several sets of simulated experiments, it was found that the reconstruction performance of IAA-ML and IAA-APES estimators is closely related to the power ratio of ground to canopy. When the ground contribution dominates, both estimators can accurately retrieve the ground scattering phase center. However, with the increase of the canopy contribution, both estimators show a degraded performance in detecting the ground phase center, especially when the canopy contribution dominates. Moreover, IAA-ML obtains a higher elevation resolution and better focusing than IAA-APES.

In addition, six P-band fully polarimetric airborne SAR images were selected to acquire the structural parameters of the forest. It was found that the results of the HH polarization are suitable for analyzing the ground contribution and the results of the HV polarization are suitable for determining the canopy contribution. Based on this, the underlying topography and forest height can be obtained. With respect to LiDAR data, the standard deviation of the estimations from the IAA-ML TomoSAR method was 2.11 m for the underlying topography and 2.80 m for the forest height, which are both less than the results of IAA-APES (2.57 m and 3.29 m, respectively). Furthermore, the estimation accuracy of IAA-ML was also found to be slightly higher than that of the SKP-beamforming technique (2.41 m vs. 2.11 m and 3.00 m vs. 2.80 m) in this case study.

In conclusion, the IAA-ML method has the advantages of a higher resolution and higher estimation accuracy than the IAA-APES approach when used with a large number of snapshots. As a result, IAA-ML is a better alternative for the forest application of TomoSAR.

In the future, we will focus on using IAA-ML in polarimetric SAR tomography (Pol-TomoSAR). In addition to the height estimation of each scatterer, the corresponding scattering mechanism will also be acquired.

## Figures and Tables

**Table 1:** Details of the IAA-ML estimator.

**Initialization**
$p_{d}=\frac{1}{{\left(a_{d}^{*}a_{d}\right)}^{2}L}\overset{L}{\underset{l=1}{\sum }}{\left|a_{d}^{*}y\left(l\right)\right|}^{2}\;\left(d=1,\cdots ,D\right)$ $R^{-1}={\left(APA^{*}\right)}^{-1}$
**Iteration**
*repeat*
1. *Adjust* $\left[i_{1},\cdots ,i_{D}\right]$ such that $p_{i_{1}}\leq \cdots \leq p_{i_{D}}$
2. $p_{i_{d}}^{previous}=p_{i_{d\;}\;}\left(d=1,\cdots ,D\right)$
3. $p_{i_{d}}=max\left(0,p_{i_{d}}^{previous}+\frac{a_{d}^{*}R^{-1}\left(\Gamma -R\right)R^{-1}a_{d}}{{\left(a_{d}^{*}R^{-1}a_{d}\right)}^{2}}\right)$ 4. $R^{-1}=R^{-1}-\frac{\left(p_{i_{d}}-p_{i_{d}}^{previous}\right)R^{-1}a_{d}a_{d}^{*}R^{-1}}{1+\left(p_{i_{d}}-p_{i_{d}}^{previous}\right)a_{d}^{*}R^{-1}a_{d}}$
*Until(convergence)*

**Table 2:** The parameters of the SETHI airborne system.

**Wavelength Polarimetric Channel**	0.7542 m (P-Band) HH + HV + VV
**Center slant range**	4905 m
**Center incidence angle**	35.0614°
**Range resolution**	1.0 m
**Azimuth resolution**	1.245 m

**Table 3:** The baseline information for the InSAR pairs.

Identifier	Acquisition Date	Baseline (m)
Tropi0402	24 August 2009	0
Tropi0403		−14.4879
Tropi0404		−30.1163
Tropi0405		−43.8343
Tropi0406		−60.0632
Tropi0407		−74.9683

**Table 4:** Mean and standard deviation of the underlying topography estimated by IAA-ML with respect to the LiDAR DTM.

TomoSAR w.r.t LiDAR	Mean	Std.
Ground (m)	1.76	2.11

**Table 5:** Mean and standard deviation of the forest height estimated by IAA-ML with respect to the LiDAR CHM.

TomoSAR w.r.t LiDAR	Mean	Std.
Forest height (m)	2.10	2.80

**Table 6:** The standard deviation of the underlying topography estimated by IAA-APES and SKP-beamforming with respect to the LiDAR data.

	TomoSAR w.r.t LiDAR	Std.
**IAA-APES**	Ground (m)	2.57
	Forest height (m)	3.29
**SKP-beamforming**	Ground (m)	2.41
	Forest height (m)	3.00
